# The Global Impact of COVID-19 on Mental Health of General Population: A Narrative Review

**DOI:** 10.7759/cureus.30627

**Published:** 2022-10-24

**Authors:** Deepak Vishwakarma, Abhay M Gaidhane, Sonali G Choudhari

**Affiliations:** 1 School of Epidemiology and Public Health, Community Medicine, Jawaharlal Nehru Medical College, Datta Meghe Institute of Medical Sciences, Wardha, IND

**Keywords:** psychological wellbeing, pandemic, sars-cov-2, covid-19, mental health

## Abstract

Mental wellness is a crucial component of happiness. A person is happy with better relationships, financial stability, good mental health, and longevity. Disinformation, stigma, ongoing isolation, and disruption of daily activities are all quite prevalent. Any of these elements may impact one's mental health. The Ministry of Health and Family Welfare has taken several steps to address COVID's mental health concerns. In addition to frontline healthcare personnel, who engage in dealing with COVID-19, the general population's mental health was also at stake due to the unprecedented and catastrophic emergency of COVID. Various keywords, including MeSH terms, were used in PubMed and Google Scholar searches. This paper was based on previously available data and article searches on how COVID-19 affected the mental health of the general population and the elements that may influence it. Quarantine and self-isolation have been found to have affected people's daily livelihoods and behaviors, leading to increased feelings of loneliness, anxiety, dejection, insomnia, risky alcohol and drug use, self-harm, and suicidal thoughts. Many of the victims of COVID-19 who were critical survivors exhibited lasting psychological harm a year after being discharged, including high anxiety levels, melancholy, and post-traumatic stress disorder. Healthcare employees too experienced significant psychological consequences due to factors such as an excessive workload or the number of hours worked, inadequate personal safety equipment, overly enthusiastic media coverage, and a sense of being under-supported. As a result of this major disaster, mental health concerns have surfaced, perhaps leading to long-term health problems, loneliness, and guilt. To reduce this deportment, global health solutions should be used, particularly while executing the isolation/quarantine and dealing with the people with fear and vulnerability. The mental health of the general population should be at the forefront of any worldwide response.

## Introduction and background

The World Health Organization classified COVID-19, an epidemic of a new coronavirus illness, as a public health emergency of international concern in January 2020 [1]. By March 2020, it had spread quickly across many continents to become a worldwide pandemic [1]. According to history, any infectious disease epidemic or pandemic is followed by a substantial mental health setback [2]. The people's psychological reactions during an infectious disease epidemic are essential in deciding how quickly the disease spreads and whether or not emotional distress and social disorder occur both during and after an outbreak [3]. The World Health Organization's China office received reports of an unidentified virus; hence the moniker novel coronavirus. COVID-19 infection was initially thought to be a viral infection. On December 31, 2019, it produced a significant spike in pneumonia cases in Wuhan, Eastern China [4]. The pandemic has impacted the global and Indian economies, with many people losing their jobs, educational uncertainty due to examination postponements, and monetary concerns due to working from home, all of which have harmed the whole strata of society physically, intellectually, and socially [5]. As a preventive approach to control the epidemic and flatten the curve to lower the peak number of illnesses and accompanying demands on health infrastructure, on March 24, India's government announced a 21-day country-wide lockdown [6].

The pandemic has had various effects on the global population [7]. Not just people with suspected COVID-19 but also people in vulnerable categories (e.g., healthcare workers, people with mental diseases, migrant workers, etc.) had to deal with mental health issues despite not being infected [8]. As a result of this global disaster, mental health concerns may arise, isolating and stigmatizing the country's most vulnerable individuals [9]. Long-term social exclusion practices, rising unemployment rates, and financial stress could lead to an unprecedented mental health crisis [10]. People's poor mental health is influenced by various things, including excessive work demands and hours, a lack of personal safety gear, overzealous media coverage, and a sense of being under-supported [11]. Since the 1918 H1N1 influenza pandemic, COVID-19 has significantly influenced the world and poses the greatest threat to public health [12]. If the COVID-19 pandemic followed the same course as the H1N1 pandemic, the psychological effects of prolonged stress on the general population and the progression of other mental health issues among the vulnerable would put further strain on the current healthcare system [13].

## Review

The paper is aimed to review how COVID-19 affected people's mental health. Articles were searched using PubMed, Google Scholar, and Science Direct. We used a combination of keywords and Medical Subject Headings (MeSH) to search for COVID's tie-up with mental health, which may help us understand the "hows" and "whys" of COVID’s relationship with mental health. The MeSH terms used were mental health, COVID-19, SARS-CoV-2, pandemic, and psychological well-being. Thirty-five articles were selected and thematically organized. The article review covered all research with free full text available through PubMed. The excluded articles were the ones that did not significantly mention how serious COVID’s implications on mental health are, the ones that did not include any mental health problems, and the ones that were not available in the English language. Articles ranging from 2020 to 2022 were used.

Discussion

COVID-19 has killed about 3.7 million people globally since the first case was discovered in the Chinese city of Wuhan. Initially, the focus was on managing immediate problems, but as the long-term effects of SARS-CoV-2 infection become increasingly apparent, it is clear that the virus has significant repercussions [14]. COVID-19, a rapidly spreading disease, has sparked a global health emergency. As a result, it could not recognize the virus's distinct concealed mental health effects, such as stress, anxiety, grief, and secondary trauma [15]. This infection may result in neuroinflammation, leading to physical problems, cognitive decline, and psychological discomfort [16-19]. Another significant problem with mental health after COVID-19 was sleep issues, particularly insomnia, seen in both the acute and chronic stages of the illness [16,20-22].

Mental wellness is a crucial component of happiness. Several suicide incidents have been observed across all socioeconomic strata during the COVID-19 era [23]. The undetermined causes of the deaths of a well-known Bollywood celebrity and an elite officer in the capital city of India have triggered a global discussion on COVID-19 mental health issues [23]. According to an article, men's and women's mental health was impacted by financial hardship. The article concludes that men's mental health may be more negatively affected by losing their jobs than women's. These findings were consistent with a significant study's findings that men are more likely than women to experience the adverse effects of unemployment [24]. However, this does not mean that women are less affected by COVID. Mental illnesses because of unemployment can be due to a sudden disruption in their schedule, disbalance in the economy leading to the permanent shutdown of reputable businesses, increased frustration, helplessness, and anxiety building inside them, making them vulnerable. In times of COVID-19, a survey from India showed that married participants had 40% lower odds of developing anxiety during the COVID-19 lockdown than unmarried participants [25]. The pandemic will eventually end, but its effects will linger for quite some time. People affected by the pandemic will experience mental health problems and other problems after the epidemic's end [26].

Separation and movement restrictions for those exposed to contagious diseases have damaged people's routine activities and everyday lives, leading to an increase in loneliness, anxiety, depression, restlessness, risky alcohol and drug use, self-harm, and suicidal thoughts and actions. Victims of domestic violence, women, and children suffer much because of restricted mobility, leaving nowhere to flee, leading to an increase in these cases worldwide [27]. Post-traumatic stress disorder (PTSD) was another widespread post-COVID-19 mental health issue, with prevalence rates ranging from 12.1% to 46.9% [28]. Anxiety and PTSD were found to be significantly more prevalent in patients admitted to the ICU than in wards, and the severity of COVID-19 correlated with the severity and prevalence of mental health symptoms [20,21,28-30]. Regarding PTSD, women are more likely than men to develop the symptoms. Still, men are more likely to experience stress and other psychological symptoms due to greater responsibilities such as family income. Lower educational levels are associated with increased PTSD symptoms and psychological distress [21]. In India, high sadness, stress, and anxiety levels have also been observed, particularly in younger persons [31].

In India, a large percentage of patients pay for their healthcare. The private sector provides a significant portion of mental healthcare, such as single chamber outpatient procedures, professional-owned mental health inpatient settings, and mental health services offered through corporate or multispecialty hospitals. A charitable organization assists in various locations. Accessing these institutions during and after COVID required payment, which made it difficult for those whose finances were impacted to get medical treatment [32]. 

Mental health implications on specific population

Children/Older People

Children and the elderly may find the abrupt and extreme changes in their daily routine distressing and challenging to cope with the situation. School closures, outside leisure activities, and the inability to socialize with classmates may harm children's mental health. In India's senior population, COVID-19 has been identified as a susceptible category [1]. This could be improved by trying to talk to them, engaging them in soulful activities, teaching them moral values, giving them a heartful diet, fostering healthy relationships with them, and trying to teach them stress management from the very beginning by teaching them ‘tapping’ their shoulders alternatively.

Student Mental Health

For many years, the mental health of the student population has been a significant concern, and the epidemic has exacerbated the problem. According to the U.K. data, slightly more than half of university students will have declining mental health by 2020. Increased psychological stress, worry, melancholy, and even post-traumatic stress disorder seem to be a worldwide epidemic in students' mental health [33]. This can be relieved by introducing interactive learning exercises daily and holding open interactive sessions for them so they can share their thoughts. This will be liberating to them and will help in lessening their stress.

The Frontline Workers

There are significant challenges that workers in community health, nursing and sanitation, police officers, doctors, and additional volunteers face globally, including making impossible decisions and working under extreme stress. Working in stressful circumstances can severely affect a person's personal and family life and mental health [1]. To reduce this, a reduction in the work hours, asking them to take short breaks within their shift, providing them protective gear, building friendly relationships with them, and making them believe that their lives are essential and an asset.

The Police Officer

Unusual jobs, difficult working conditions, and the ambiguity of the police role have already been cited as sources of occupational stress among Indian police officers. They have been linked to workplace stress and burnout. Fears of contracting the virus in the community and at work may also be a source of anxiety for cops [34]. Providing them a day off when work is challenging and noticing their behavior to check for signs of frustration or anxiety. In the department, they must be provided with training to cope with extreme stress. They must be encouraged to take proper sleep.

Mental Health Issues After Covid-19 Recovery

Issues with mental health following COVID-19 recovery: Delirium is common in patients who require hospitalization following COVID-19 recovery during the acute stage of the illness [35]. Although the long-term psychological problems in this group of patients are unknown, they could be similar to previous coronavirus outbreaks like SARS and middle east respiratory syndrome (MERS), which were linked to more excellent rates of post-traumatic stress disorder, depression, and anxiety [35]. (Table 1) shows the possible causes of mental health problems and their suggested solutions for mental health diseases [2].

**Table 1:** Possible causes of mental health problems and management solutions for mental health diseases associated with COVID-19 in diverse at-risk populations. Adapted from [2].

Population Group at Risk	Factors Affecting Mental Health Problems During the COVID-19 Epidemic	Mental Health and COVID-19 Problem Recommended Therapeutic Strategies
Children	Changes in one's daily routine: Schools will be closed, and outdoor recreational activities will be limited: Not being able to see their mates and classmates.	They were setting limits on screen time to limit exposure to negative news while maintaining the accuracy of information. Engage in indoor creative and intellectual pursuits. Recognizing a kid's emotional requirements and controlling their anxiety. Establishing a home learning schedule is one method people keep in touch with their friends. Or by using email, social media, or text messaging. Creating a learning routine at home.
Geriatric Population	The elderly have underlying comorbid problems that make it difficult for them to access online or telemedicine services for healthcare because of difficulties using smartphones or computers, which interferes with their regular healthcare. These difficulties include a problem with day-to-day activities for those living alone, feeling isolated socially due to lockdown, and difficulty in using smartphones or computers, making it difficult for them to access online or telemedicine services for healthcare.	Distributing information politely that is clear, concise, and required—reassurance and support for the most helpless. Carefully address mental health issues by involving family members and support personnel, connecting with family far away, and participating in leisure activities. Spending time away from the news and ensuring there are enough prescription meds for those in need to treat any insecurities.
Migrant Workers	Less comfortable in their temporary surroundings, worried about their relatives who reside elsewhere, and suffering financially and economically loss as daily wage laborers.	They are individualizing migrant workers' treatment by treating them with respect, empathy, and compassion and assuring that economic assistance and assurance are provided respectfully. They are stressing the importance of staying and ensuring their mental and physical support while separated from their families. Consistent, methodical security, effective counseling, and basic needs provision.
Frontline Workers	There is a possibility that someone could catch the disease while caring for others, and this could lead to fear of infecting their family members. As the healthcare system's load grows, this could lead to more stress.	To all the frontline workers, by providing proper and protective ergonomic gear, we can help prevent infection. Providing incentives to them and their families for risking their lives to safeguard others. Ensuring workplace respect and safety. Recognizing and applauding their selfless efforts, as well as appropriately paying them back.
Individuals with COVID-19, Contacts, Survivors, and Family	The sensation of being the source of the disease spreading to others. Complete isolation from family and friends. Discrimination leads to emotional distress.	Addressing the loss and anguish experienced by COVID-19 patients and their families, self-help platforms are being developed. Providing support to those who have lost a loved one due to the pandemic. Recognizing survivors and offering mental and physical support to them at their detention centers or hospitals.
Individuals With Active Mental Diseases	Triggers include being confined in one's own house, quarantine, and isolation. Continuum of care may be affected as tele-counseling sessions may not be as effective as face-to-face sessions. Non-availability of entertainment factors. A change in the daily routine of people with pre-existing mental illness.	Correctly modifying their therapy sessions through telemedicine and video conferencing to assist patients in coping with the pandemic and their underlying condition. It is recommended that family members be involved in the care and attention of their loved one who is experiencing a mental health issue.

Table 2 shows the initiatives taken by different states and organizational bodies [2].

**Table 2:** Different states and institutional/organizational bodies have taken various mental health interventions or initiatives Adapted from [2].

Institution/State	Initiative	Description
Maharashtra's government	The government of Maharashtra has set up two helplines: 1800120820050 and 18001024040.	The Mumbai municipal government created this helpline number in partnership with Mpower, a program of the Aditya Birla Education Trust, which helped manage tension throughout the lockout (Mumbai: During the coronavirus lockdown, the helpline for mental health is flooded with calls – India News, 2020).
Administration of Telangana	The government of Telangana established 108 hotlines for mental health counseling.	The State Government has chosen to use the 108 helplines to offer crucial throughout the lengthy lockdown. And they were provided comprehensive public counseling (The New Indian Express 2020: The government will employ the 108 helplines for mental health counseling).
Administration of Madhya Pradesh	It has been agreed to resurrect the Madhya Pradesh initiative of the department of happiness.	Anand department, which will collaborate with social workers to create appropriate activities for hospital patients. They will receive videos, music, and light amusement, which will help COVID-19 sufferers feel less stressed.
Administration of Kerala	During the shutdown, the elderly are given special attention by Kudumbashree.	While the closure of COVID-19, this campaign assured the well-being of the state's elderly residents by interacting with families and implementing several self-assurance-enhancing techniques. The program's primary goal is to improve the elderly's mental health and confidence by providing appropriate Information Education Communication (IEC) materials. During the closure, Kudumbashree makes an extra effort to help the elderly.
Odisha's government	The Department of Health and Family Welfare in Odisha has released guidelines to assist the state's most vulnerable citizens. The continuum of care and mental health issue have separate Telemedicine helplines.	To promote and defend public mental health and vulnerable populations, these principles are meant to be used as public awareness, education, and communication instruments (health department, 2020).
Bengaluru's NIMHANS	With the cooperation of NIMHANS, the government launches a toll-free helpline for mental health issues.	For anyone facing mental health concerns due to the current nationwide lockdown to stem the spread of the coronavirus. The government has set up a free helpline at 08046110007. During the lockdown, the government set up a telephone number for the mental health helpline (The Economic Times, 2021).
Hyderabad's center for mental health	Hyderabad launches an all-India helpline to cope with the mental strain of the lockdown.	Over 800 people have sought outpatient treatment at Hyderabad's Erragadda’s Institute of Mental Health for various issues in the last two weeks, despite the lockdown and travel restrictions. The Indian Express reported in 2020, "Hyderabad establishes an all-India helpline with curfew making people anxious Coronavirus pandemic news," that approximately 170 patients were admitted.
Tamil Nadu Psychology Association is a non-profit organization dedicated to promoting psychology.	Tamil Nadu offers telepsychiatry counseling.	More than 60 psychologists from the nation were collected by the Chennai-based mastermind foundation, a mental health organization, to provide 24/7 counseling. (Helpful numbers in these times - The Hindu, 2020) 11 languages.

Limitations 

One of the limitations of this review is that we looked at mental health as a general issue rather than taking into account all the minor and large issues that it covers. Mental health is an umbrella terminology and needs more acknowledgment everywhere.

## Conclusions

Significant levels of psychological anguish are linked to the COVID-19 pandemic. The public health focus should be on reducing the risk that COVID-19 has on mental health. People are still affected by COVID-related illnesses to date, in addition to when it was at its worst. Everyone is affected, whether they are migrant workers or those with white-collar professions. Depression, anxiety, fear, stress, PTSD, insomnia, hypersomnia, mood disorders, autophobia, and many other disorders were prevalent. When pandemic or epidemic strikes, there is no simple solution to come out of it. The current focus on the COVID-19 virus's global spread is likely to deflect attention from the emotional impacts of the pandemic on those affected and the general public. This global catastrophe has raised mental issues that could result in long-term health problems, loneliness, and guilt. Global health solutions should be implemented, especially concerning the prevalence of isolation/quarantine, fear, and susceptibility among the general people, to reduce psychological strain. Patients and the general public's mental health should be at the forefront of any global response. Many interventional therapies like group therapy, cognitive behavioral therapy (CBT), guided self-help, counseling, behavioral activation, interpersonal therapy, eye movement and desensitization and reprocessing, and mindfulness-based cognitive therapy should focus on implementation. Apart from this, press and social media information should be adequately regulated and monitored, and community participation should be encouraged. Collective efforts must be taken everywhere, seeing how major mental health problems are. They must be faced boldly since it involves future generations who will be impacted if mental health continues to grip our minds, body, and soul.
